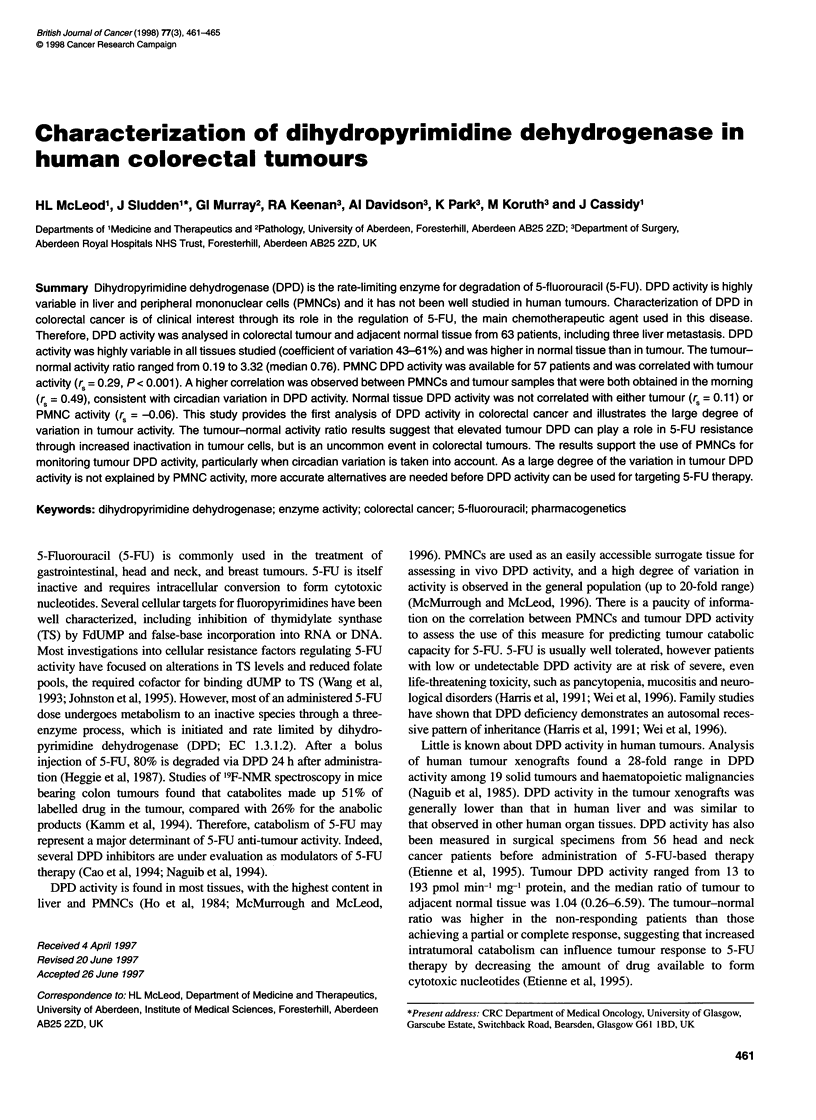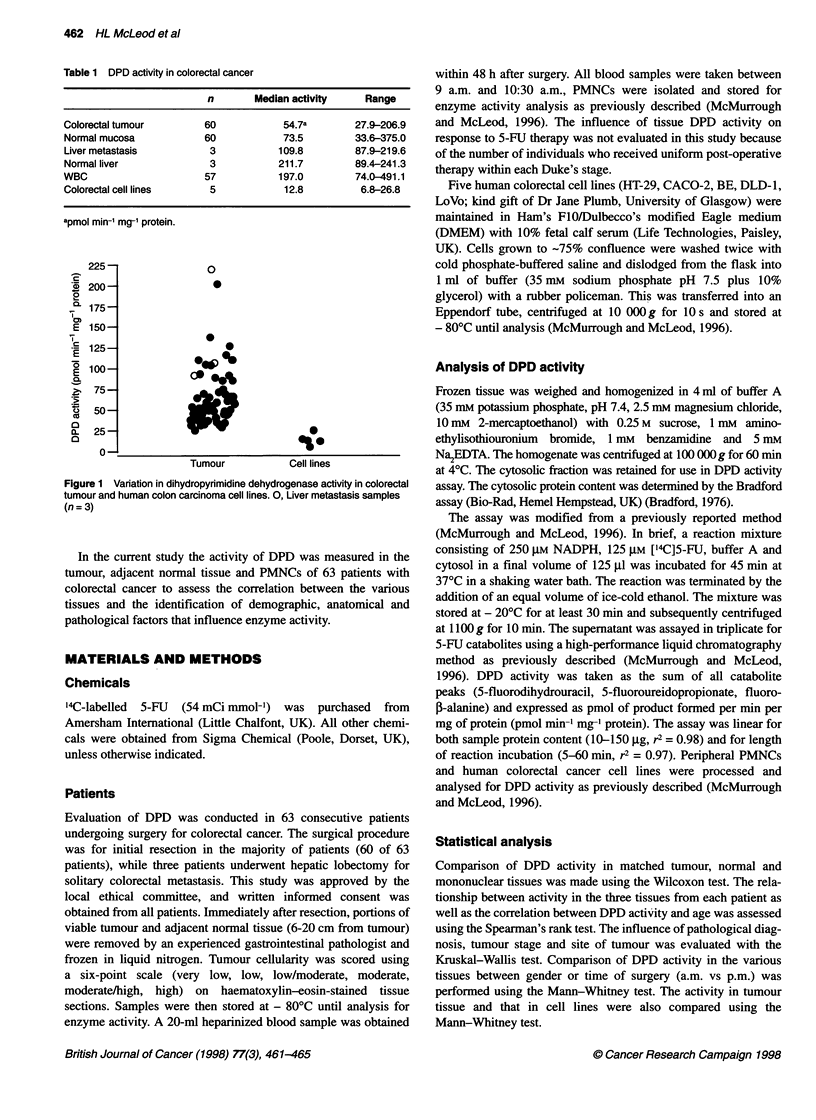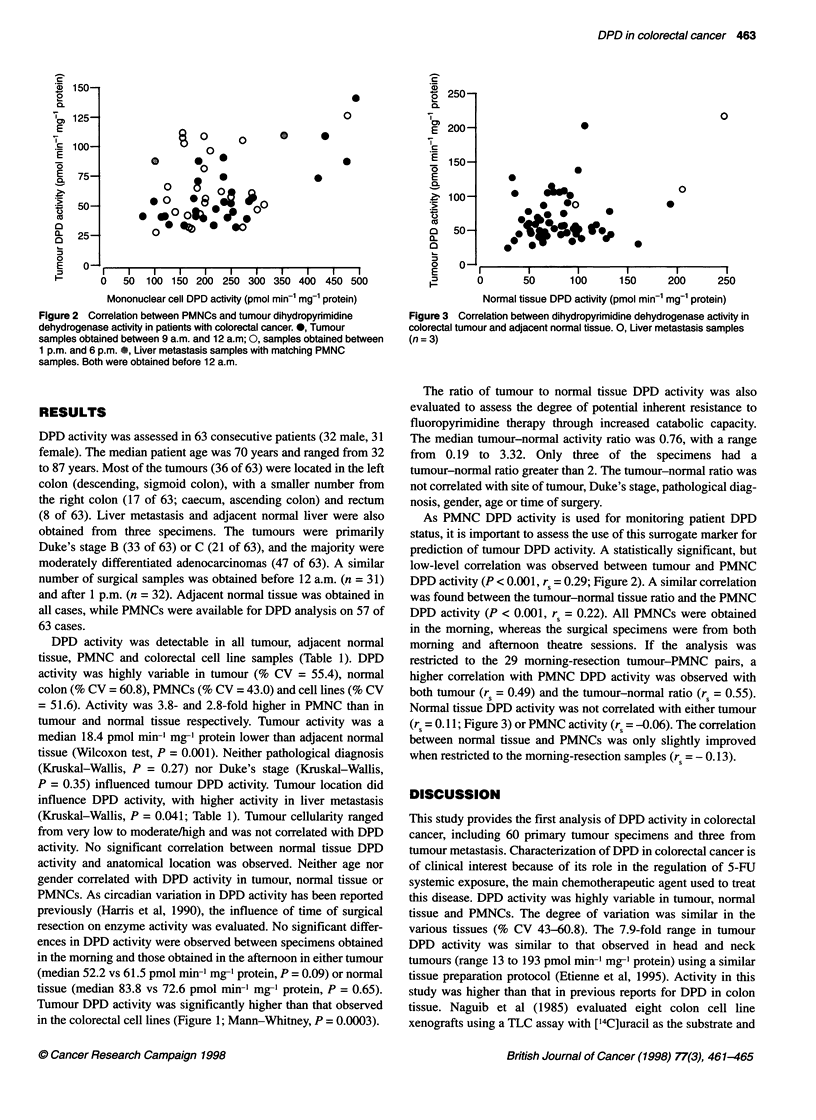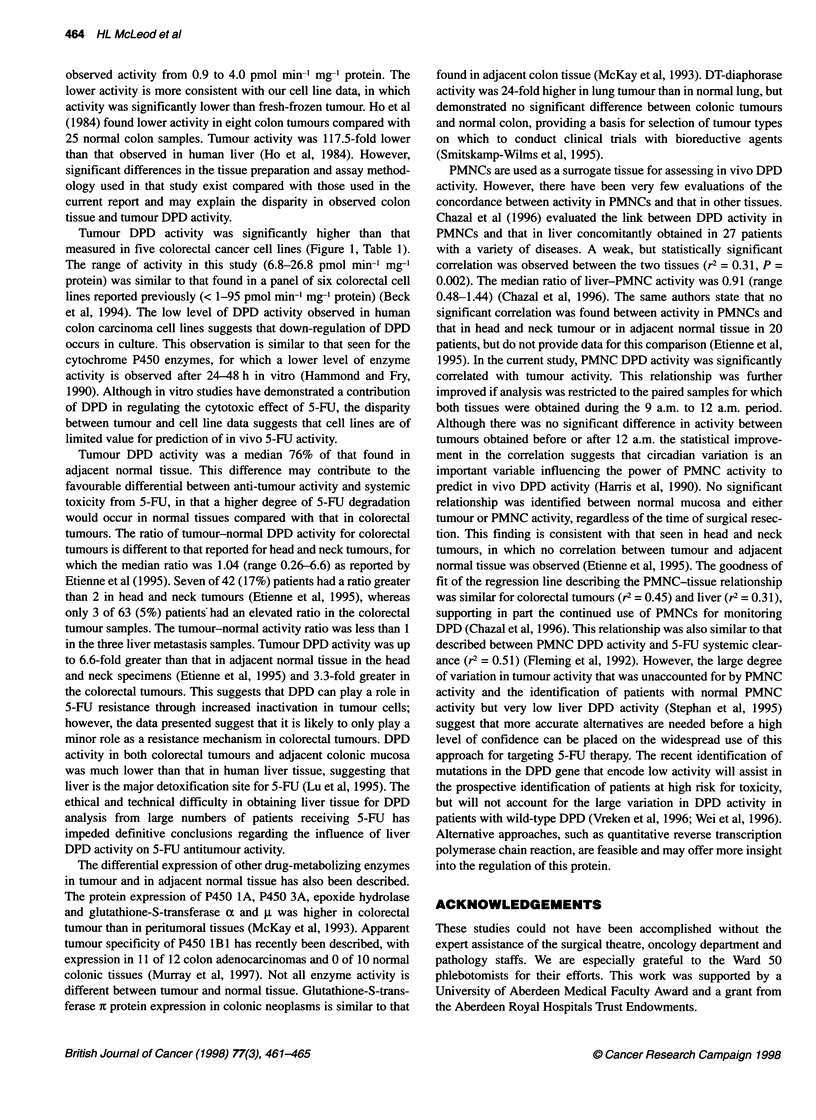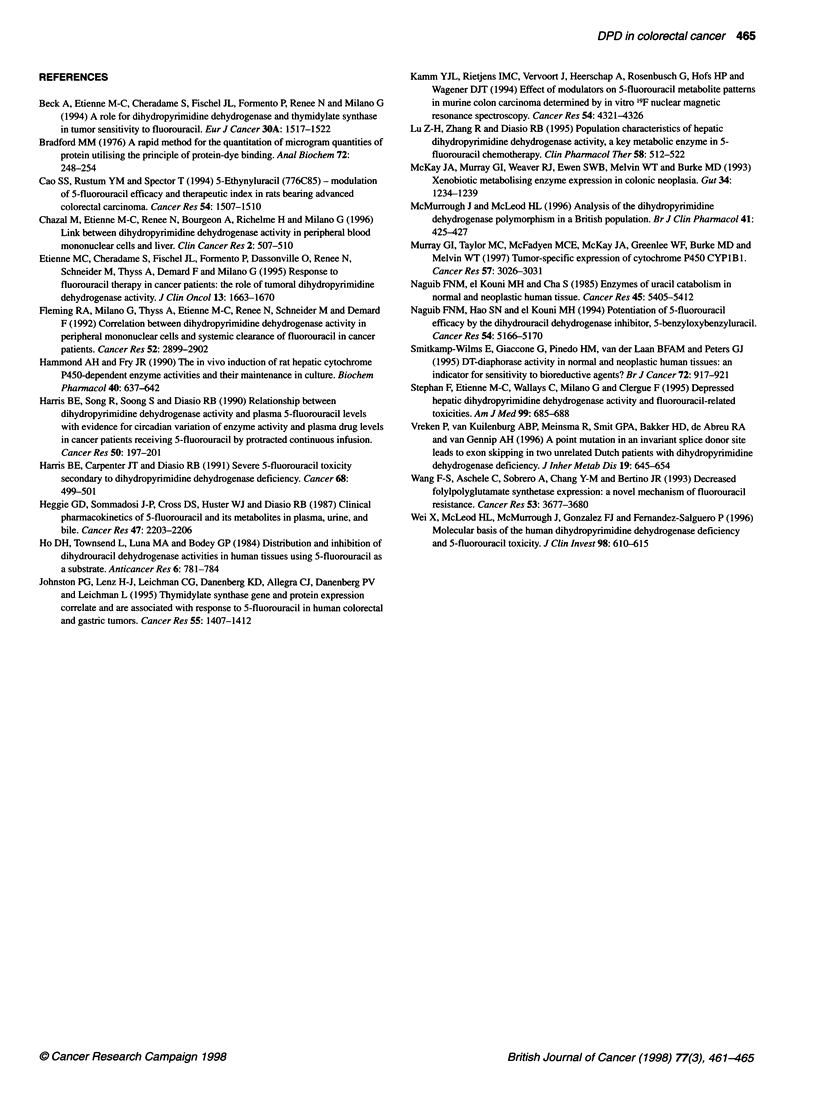# Characterization of dihydropyrimidine dehydrogenase in human colorectal tumours.

**DOI:** 10.1038/bjc.1998.73

**Published:** 1998

**Authors:** H. L. McLeod, J. Sludden, G. I. Murray, R. A. Keenan, A. I. Davidson, K. Park, M. Koruth, J. Cassidy

**Affiliations:** Department of Medicine, University of Aberdeen, Foresterhill, UK.

## Abstract

Dihydropyrimidine dehydrogenase (DPD) is the rate-limiting enzyme for degradation of 5-fluorouracil (5-FU). DPD activity is highly variable in liver and peripheral mononuclear cells (PMNCs) and it has not been well studied in human tumours. Characterization of DPD in colorectal cancer is of clinical interest through its role in the regulation of 5-FU, the main chemotherapeutic agent used in this disease. Therefore, DPD activity was analysed in colorectal tumour and adjacent normal tissue from 63 patients, including three liver metastasis. DPD activity was highly variable in all tissues studied (coefficient of variation 43-61%) and was higher in normal tissue than in tumour. The tumour-normal activity ratio ranged from 0.19 to 3.32 (median 0.76). PMNC DPD activity was available for 57 patients and was correlated with tumour activity (r(s) = 0.29, P < 0.001). A higher correlation was observed between PMNCs and tumour samples that were both obtained in the morning (r(s) = 0.49), consistent with circadian variation in DPD activity. Normal tissue DPD activity was not correlated with either tumour (r(s) = 0.11) or PMNC activity (r(s) = -0.06). This study provides the first analysis of DPD activity in colorectal cancer and illustrates the large degree of variation in tumour activity. The tumour-normal activity ratio results suggest that elevated tumour DPD can play a role in 5-FU resistance through increased inactivation in tumour cells, but is an uncommon event in colorectal tumours. The results support the use of PMNCs for monitoring tumour DPD activity, particularly when circadian variation is taken into account. As a large degree of the variation in tumour DPD activity is not explained by PMNC activity, more accurate alternatives are needed before DPD activity can be used for targeting 5-FU therapy.


British Journal of Cancer (1998) 77(3), 461–465
© 1998 Cancer Research Campaign

### Characterization of dihydropyrimidine dehydrogenase in human colorectal tumours

**HL McLeod[1], J Sludden[1*], GI Murray[2], RA Keenan[3], AI Davidson[3], K Park[3], M Koruth[3] and J Cassidy[1]**

Departments of [1]Medicine and Therapeutics and [2]Pathology, University of Aberdeen, Foresterhill, Aberdeen AB25 2ZD; [3]Department of Surgery, Aberdeen Royal Hospitals NHS Trust, Foresterhill, Aberdeen AB25 2ZD, UK

**Summary** Dihydropyrimidine dehydrogenase (DPD) is the rate-limiting enzyme for degradation of 5-fluorouracil (5-FU). DPD activity is highly variable in liver and peripheral mononuclear cells (PMNCs) and it has not been well studied in human tumours. Characterization of DPD in colorectal cancer is of clinical interest through its role in the regulation of 5-FU, the main chemotherapeutic agent used in this disease. Therefore, DPD activity was analysed in colorectal tumour and adjacent normal tissue from 63 patients, including three liver metastasis. DPD activity was highly variable in all tissues studied (coefficient of variation 43–61%) and was higher in normal tissue than in tumour. The tumour–normal activity ratio ranged from 0.19 to 3.32 (median 0.76). PMNC DPD activity was available for 57 patients and was correlated with tumour activity ($r_s$ = 0.29, $P$ < 0.001). A higher correlation was observed between PMNCs and tumour samples that were both obtained in the morning ($r_s$ = 0.49), consistent with circadian variation in DPD activity. Normal tissue DPD activity was not correlated with either tumour ($r_s$ = 0.11) or PMNC activity ($r_s$ = –0.06). This study provides the first analysis of DPD activity in colorectal cancer and illustrates the large degree of variation in tumour activity. The tumour–normal activity ratio results suggest that elevated tumour DPD can play a role in 5-FU resistance through increased inactivation in tumour cells, but is an uncommon event in colorectal tumours. The results support the use of PMNCs for monitoring tumour DPD activity, particularly when circadian variation is taken into account. As a large degree of the variation in tumour DPD activity is not explained by PMNC activity, more accurate alternatives are needed before DPD activity can be used for targeting 5-FU therapy.

**Keywords:** dihydropyrimidine dehydrogenase; enzyme activity; colorectal cancer; 5-fluorouracil; pharmacogenetics

5-Fluorouracil (5-FU) is commonly used in the treatment of gastrointestinal, head and neck, and breast tumours. 5-FU is itself inactive and requires intracellular conversion to form cytotoxic nucleotides. Several cellular targets for fluoropyrimidines have been well characterized, including inhibition of thymidylate synthase (TS) by FdUMP and false-base incorporation into RNA or DNA. Most investigations into cellular resistance factors regulating 5-FU activity have focused on alterations in TS levels and reduced folate pools, the required cofactor for binding dUMP to TS (Wang et al, 1993; Johnston et al, 1995). However, most of an administered 5-FU dose undergoes metabolism to an inactive species through a three-enzyme process, which is initiated and rate limited by dihydropyrimidine dehydrogenase (DPD; EC 1.3.1.2). After a bolus injection of 5-FU, 80% is degraded via DPD 24 h after administration (Heggie et al, 1987). Studies of $^{19}$F-NMR spectroscopy in mice bearing colon tumours found that catabolites made up 51% of labelled drug in the tumour, compared with 26% for the anabolic products (Kamm et al, 1994). Therefore, catabolism of 5-FU may represent a major determinant of 5-FU anti-tumour activity. Indeed, several DPD inhibitors are under evaluation as modulators of 5-FU therapy (Cao et al, 1994; Naguib et al, 1994).

DPD activity is found in most tissues, with the highest content in liver and PMNCs (Ho et al, 1984; McMurrough and McLeod, 1996). PMNCs are used as an easily accessible surrogate tissue for assessing in vivo DPD activity, and a high degree of variation in activity is observed in the general population (up to 20-fold range) (McMurrough and McLeod, 1996). There is a paucity of information on the correlation between PMNCs and tumour DPD activity to assess the use of this measure for predicting tumour catabolic capacity for 5-FU. 5-FU is usually well tolerated, however patients with low or undetectable DPD activity are at risk of severe, even life-threatening toxicity, such as pancytopenia, mucositis and neurological disorders (Harris et al, 1991; Wei et al, 1996). Family studies have shown that DPD deficiency demonstrates an autosomal recessive pattern of inheritance (Harris et al, 1991; Wei et al, 1996).

Little is known about DPD activity in human tumours. Analysis of human tumour xenografts found a 28-fold range in DPD activity among 19 solid tumours and haematopoietic malignancies (Naguib et al, 1985). DPD activity in the tumour xenografts was generally lower than that in human liver and was similar to that observed in other human organ tissues. DPD activity has also been measured in surgical specimens from 56 head and neck cancer patients before administration of 5-FU-based therapy (Etienne et al, 1995). Tumour DPD activity ranged from 13 to 193 pmol min$^{-1}$ mg$^{-1}$ protein, and the median ratio of tumour to adjacent normal tissue was 1.04 (0.26–6.59). The tumour–normal ratio was higher in the non-responding patients than those achieving a partial or complete response, suggesting that increased intratumoral catabolism can influence tumour response to 5-FU therapy by decreasing the amount of drug available to form cytotoxic nucleotides (Etienne et al, 1995).

*Received 4 April 1997*
*Revised 20 June 1997*
*Accepted 26 June 1997*

*Correspondence to:* HL McLeod, Department of Medicine and Therapeutics, University of Aberdeen, Institute of Medical Sciences, Foresterhill, Aberdeen AB25 2ZD, UK

**Present address:* CRC Department of Medical Oncology, University of Glasgow, Garscube Estate, Switchback Road, Bearsden, Glasgow G61 1BD, UK

**Table 1** DPD activity in colorectal cancer

| | *n* | Median activity | Range |
|---|---|---|---|
| Colorectal tumour | 60 | 54.7[a] | 27.9–206.9 |
| Normal mucosa | 60 | 73.5 | 33.6–375.0 |
| Liver metastasis | 3 | 109.8 | 87.9–219.6 |
| Normal liver | 3 | 211.7 | 89.4–241.3 |
| WBC | 57 | 197.0 | 74.0–491.1 |
| Colorectal cell lines | 5 | 12.8 | 6.8–26.8 |

[a]pmol min$^{-1}$ mg$^{-1}$ protein.

**Figure 1** Variation in dihydropyrimidine dehydrogenase activity in colorectal tumour and human colon carcinoma cell lines. O, Liver metastasis samples ($n$ = 3)

In the current study the activity of DPD was measured in the tumour, adjacent normal tissue and PMNCs of 63 patients with colorectal cancer to assess the correlation between the various tissues and the identification of demographic, anatomical and pathological factors that influence enzyme activity.

### MATERIALS AND METHODS

### Chemicals

$^{14}$C-labelled 5-FU (54 mCi mmol$^{-1}$) was purchased from Amersham International (Little Chalfont, UK). All other chemicals were obtained from Sigma Chemical (Poole, Dorset, UK), unless otherwise indicated.

### Patients

Evaluation of DPD was conducted in 63 consecutive patients undergoing surgery for colorectal cancer. The surgical procedure was for initial resection in the majority of patients (60 of 63 patients), while three patients underwent hepatic lobectomy for solitary colorectal metastasis. This study was approved by the local ethical committee, and written informed consent was obtained from all patients. Immediately after resection, portions of viable tumour and adjacent normal tissue (6-20 cm from tumour) were removed by an experienced gastrointestinal pathologist and frozen in liquid nitrogen. Tumour cellularity was scored using a six-point scale (very low, low, low/moderate, moderate, moderate/high, high) on haematoxylin–eosin-stained tissue sections. Samples were then stored at – 80°C until analysis for enzyme activity. A 20-ml heparinized blood sample was obtained within 48 h after surgery. All blood samples were taken between 9 a.m. and 10:30 a.m., PMNCs were isolated and stored for enzyme activity analysis as previously described (McMurrough and McLeod, 1996). The influence of tissue DPD activity on response to 5-FU therapy was not evaluated in this study because of the number of individuals who received uniform post-operative therapy within each Duke's stage.

Five human colorectal cell lines (HT-29, CACO-2, BE, DLD-1, LoVo; kind gift of Dr Jane Plumb, University of Glasgow) were maintained in Ham's F10/Dulbecco's modified Eagle medium (DMEM) with 10% fetal calf serum (Life Technologies, Paisley, UK). Cells grown to ~75% confluence were washed twice with cold phosphate-buffered saline and dislodged from the flask into 1 ml of buffer (35 mM sodium phosphate pH 7.5 plus 10% glycerol) with a rubber policeman. This was transferred into an Eppendorf tube, centrifuged at 10 000 *g* for 10 s and stored at – 80°C until analysis (McMurrough and McLeod, 1996).

### Analysis of DPD activity

Frozen tissue was weighed and homogenized in 4 ml of buffer A (35 mM potassium phosphate, pH 7.4, 2.5 mM magnesium chloride, 10 mM 2-mercaptoethanol) with 0.25 M sucrose, 1 mM amino-ethylisothiouronium bromide, 1 mM benzamidine and 5 mM $Na_2EDTA$. The homogenate was centrifuged at 100 000 *g* for 60 min at 4°C. The cytosolic fraction was retained for use in DPD activity assay. The cytosolic protein content was determined by the Bradford assay (Bio-Rad, Hemel Hempstead, UK) (Bradford, 1976).

The assay was modified from a previously reported method (McMurrough and McLeod, 1996). In brief, a reaction mixture consisting of 250 μM NADPH, 125 μM [$^{14}$C]5-FU, buffer A and cytosol in a final volume of 125 μl was incubated for 45 min at 37°C in a shaking water bath. The reaction was terminated by the addition of an equal volume of ice-cold ethanol. The mixture was stored at – 20°C for at least 30 min and subsequently centrifuged at 1100 *g* for 10 min. The supernatant was assayed in triplicate for 5-FU catabolites using a high-performance liquid chromatography method as previously described (McMurrough and McLeod, 1996). DPD activity was taken as the sum of all catabolite peaks (5-fluorodihydrouracil, 5-fluoroureidopropionate, fluoro-β-alanine) and expressed as pmol of product formed per min per mg of protein (pmol min$^{-1}$ mg$^{-1}$ protein). The assay was linear for both sample protein content (10–150 μg, $r^2$ = 0.98) and for length of reaction incubation (5–60 min, $r^2$ = 0.97). Peripheral PMNCs and human colorectal cancer cell lines were processed and analysed for DPD activity as previously described (McMurrough and McLeod, 1996).

### Statistical analysis

Comparison of DPD activity in matched tumour, normal and mononuclear tissues was made using the Wilcoxon test. The relationship between activity in the three tissues from each patient as well as the correlation between DPD activity and age was assessed using the Spearman's rank test. The influence of pathological diagnosis, tumour stage and site of tumour was evaluated with the Kruskal–Wallis test. Comparison of DPD activity in the various tissues between gender or time of surgery (a.m. vs p.m.) was performed using the Mann–Whitney test. The activity in tumour tissue and that in cell lines were also compared using the Mann–Whitney test.

© Cancer Research Campaign 1998

**Figure 2** Correlation between PMNCs and tumour dihydropyrimidine dehydrogenase activity in patients with colorectal cancer. ●, Tumour samples obtained between 9 a.m. and 12 a.m; ○, samples obtained between 1 p.m. and 6 p.m. ◉, Liver metastasis samples with matching PMNC samples. Both were obtained before 12 a.m.

**Figure 3** Correlation between dihydropyrimidine dehydrogenase activity in colorectal tumour and adjacent normal tissue. O, Liver metastasis samples ($n$ = 3)

### RESULTS

DPD activity was assessed in 63 consecutive patients (32 male, 31 female). The median patient age was 70 years and ranged from 32 to 87 years. Most of the tumours (36 of 63) were located in the left colon (descending, sigmoid colon), with a smaller number from the right colon (17 of 63; caecum, ascending colon) and rectum (8 of 63). Liver metastasis and adjacent normal liver were also obtained from three specimens. The tumours were primarily Duke's stage B (33 of 63) or C (21 of 63), and the majority were moderately differentiated adenocarcinomas (47 of 63). A similar number of surgical samples was obtained before 12 a.m. ($n$ = 31) and after 1 p.m. ($n$ = 32). Adjacent normal tissue was obtained in all cases, while PMNCs were available for DPD analysis on 57 of 63 cases.

DPD activity was detectable in all tumour, adjacent normal tissue, PMNC and colorectal cell line samples (Table 1). DPD activity was highly variable in tumour (% CV = 55.4), normal colon (% CV = 60.8), PMNCs (% CV = 43.0) and cell lines (% CV = 51.6). Activity was 3.8- and 2.8-fold higher in PMNC than in tumour and normal tissue respectively. Tumour activity was a median 18.4 pmol min$^{-1}$ mg$^{-1}$ protein lower than adjacent normal tissue (Wilcoxon test, $P$ = 0.001). Neither pathological diagnosis (Kruskal–Wallis, $P$ = 0.27) nor Duke's stage (Kruskal–Wallis, $P$ = 0.35) influenced tumour DPD activity. Tumour location did influence DPD activity, with higher activity in liver metastasis (Kruskal–Wallis, $P$ = 0.041; Table 1). Tumour cellularity ranged from very low to moderate/high and was not correlated with DPD activity. No significant correlation between normal tissue DPD activity and anatomical location was observed. Neither age nor gender correlated with DPD activity in tumour, normal tissue or PMNCs. As circadian variation in DPD activity has been reported previously (Harris et al, 1990), the influence of time of surgical resection on enzyme activity was evaluated. No significant differences in DPD activity were observed between specimens obtained in the morning and those obtained in the afternoon in either tumour (median 52.2 vs 61.5 pmol min$^{-1}$ mg$^{-1}$ protein, $P$ = 0.09) or normal tissue (median 83.8 vs 72.6 pmol min$^{-1}$ mg$^{-1}$ protein, $P$ = 0.65). Tumour DPD activity was significantly higher than that observed in the colorectal cell lines (Figure 1; Mann–Whitney, $P$ = 0.0003).

The ratio of tumour to normal tissue DPD activity was also evaluated to assess the degree of potential inherent resistance to fluoropyrimidine therapy through increased catabolic capacity. The median tumour–normal activity ratio was 0.76, with a range from 0.19 to 3.32. Only three of the specimens had a tumour–normal ratio greater than 2. The tumour–normal ratio was not correlated with site of tumour, Duke's stage, pathological diagnosis, gender, age or time of surgery.

As PMNC DPD activity is used for monitoring patient DPD status, it is important to assess the use of this surrogate marker for prediction of tumour DPD activity. A statistically significant, but low-level correlation was observed between tumour and PMNC DPD activity ($P < 0.001$, $r_s = 0.29$; Figure 2). A similar correlation was found between the tumour–normal tissue ratio and the PMNC DPD activity ($P < 0.001$, $r_s = 0.22$). All PMNCs were obtained in the morning, whereas the surgical specimens were from both morning and afternoon theatre sessions. If the analysis was restricted to the 29 morning-resection tumour–PMNC pairs, a higher correlation with PMNC DPD activity was observed with both tumour ($r_s = 0.49$) and the tumour–normal ratio ($r_s = 0.55$). Normal tissue DPD activity was not correlated with either tumour ($r_s = 0.11$; Figure 3) or PMNC activity ($r_s = -0.06$). The correlation between normal tissue and PMNCs was only slightly improved when restricted to the morning-resection samples ($r_s = -0.13$).

### DISCUSSION

This study provides the first analysis of DPD activity in colorectal cancer, including 60 primary tumour specimens and three from tumour metastasis. Characterization of DPD in colorectal cancer is of clinical interest because of its role in the regulation of 5-FU systemic exposure, the main chemotherapeutic agent used to treat this disease. DPD activity was highly variable in tumour, normal tissue and PMNCs. The degree of variation was similar in the various tissues (% CV 43–60.8). The 7.9-fold range in tumour DPD activity was similar to that observed in head and neck tumours (range 13 to 193 pmol min$^{-1}$ mg$^{-1}$ protein) using a similar tissue preparation protocol (Etienne et al, 1995). Activity in this study was higher than that in previous reports for DPD in colon tissue. Naguib et al (1985) evaluated eight colon cell line xenografts using a TLC assay with [$^{14}$C]uracil as the substrate and

© Cancer Research Campaign 1998

observed activity from 0.9 to 4.0 pmol min$^{-1}$ mg$^{-1}$ protein. The lower activity is more consistent with our cell line data, in which activity was significantly lower than fresh-frozen tumour. Ho et al (1984) found lower activity in eight colon tumours compared with 25 normal colon samples. Tumour activity was 117.5-fold lower than that observed in human liver (Ho et al, 1984). However, significant differences in the tissue preparation and assay methodology used in that study exist compared with those used in the current report and may explain the disparity in observed colon tissue and tumour DPD activity.

Tumour DPD activity was significantly higher than that measured in five colorectal cancer cell lines (Figure 1, Table 1). The range of activity in this study (6.8–26.8 pmol min$^{-1}$ mg$^{-1}$ protein) was similar to that found in a panel of six colorectal cell lines reported previously (< 1–95 pmol min$^{-1}$ mg$^{-1}$ protein) (Beck et al, 1994). The low level of DPD activity observed in human colon carcinoma cell lines suggests that down-regulation of DPD occurs in culture. This observation is similar to that seen for the cytochrome P450 enzymes, for which a lower level of enzyme activity is observed after 24–48 h in vitro (Hammond and Fry, 1990). Although in vitro studies have demonstrated a contribution of DPD in regulating the cytotoxic effect of 5-FU, the disparity between tumour and cell line data suggests that cell lines are of limited value for prediction of in vivo 5-FU activity.

Tumour DPD activity was a median 76% of that found in adjacent normal tissue. This difference may contribute to the favourable differential between anti-tumour activity and systemic toxicity from 5-FU, in that a higher degree of 5-FU degradation would occur in normal tissues compared with that in colorectal tumours. The ratio of tumour–normal DPD activity for colorectal tumours is different to that reported for head and neck tumours, for which the median ratio was 1.04 (range 0.26–6.6) as reported by Etienne et al (1995). Seven of 42 (17%) patients had a ratio greater than 2 in head and neck tumours (Etienne et al, 1995), whereas only 3 of 63 (5%) patients had an elevated ratio in the colorectal tumour samples. The tumour–normal activity ratio was less than 1 in the three liver metastasis samples. Tumour DPD activity was up to 6.6-fold greater than that in adjacent normal tissue in the head and neck specimens (Etienne et al, 1995) and 3.3-fold greater in the colorectal tumours. This suggests that DPD can play a role in 5-FU resistance through increased inactivation in tumour cells; however, the data presented suggest that it is likely to only play a minor role as a resistance mechanism in colorectal tumours. DPD activity in both colorectal tumours and adjacent colonic mucosa was much lower than that in human liver tissue, suggesting that liver is the major detoxification site for 5-FU (Lu et al, 1995). The ethical and technical difficulty in obtaining liver tissue for DPD analysis from large numbers of patients receiving 5-FU has impeded definitive conclusions regarding the influence of liver DPD activity on 5-FU antitumour activity.

The differential expression of other drug-metabolizing enzymes in tumour and in adjacent normal tissue has also been described. The protein expression of P450 1A, P450 3A, epoxide hydrolase and glutathione-S-transferase $\alpha$ and $\mu$ was higher in colorectal tumour than in peritumoral tissues (McKay et al, 1993). Apparent tumour specificity of P450 1B1 has recently been described, with expression in 11 of 12 colon adenocarcinomas and 0 of 10 normal colonic tissues (Murray et al, 1997). Not all enzyme activity is different between tumour and normal tissue. Glutathione-S-transferase $\pi$ protein expression in colonic neoplasms is similar to that found in adjacent colon tissue (McKay et al, 1993). DT-diaphorase activity was 24-fold higher in lung tumour than in normal lung, but demonstrated no significant difference between colonic tumours and normal colon, providing a basis for selection of tumour types on which to conduct clinical trials with bioreductive agents (Smitskamp-Wilms et al, 1995).

PMNCs are used as a surrogate tissue for assessing in vivo DPD activity. However, there have been very few evaluations of the concordance between activity in PMNCs and that in other tissues. Chazal et al (1996) evaluated the link between DPD activity in PMNCs and that in liver concomitantly obtained in 27 patients with a variety of diseases. A weak, but statistically significant correlation was observed between the two tissues ($r^2 = 0.31$, $P = 0.002$). The median ratio of liver–PMNC activity was 0.91 (range 0.48–1.44) (Chazal et al, 1996). The same authors state that no significant correlation was found between activity in PMNCs and that in head and neck tumour or in adjacent normal tissue in 20 patients, but do not provide data for this comparison (Etienne et al, 1995). In the current study, PMNC DPD activity was significantly correlated with tumour activity. This relationship was further improved if analysis was restricted to the paired samples for which both tissues were obtained during the 9 a.m. to 12 a.m. period. Although there was no significant difference in activity between tumours obtained before or after 12 a.m. the statistical improvement in the correlation suggests that circadian variation is an important variable influencing the power of PMNC activity to predict in vivo DPD activity (Harris et al, 1990). No significant relationship was identified between normal mucosa and either tumour or PMNC activity, regardless of the time of surgical resection. This finding is consistent with that seen in head and neck tumours, in which no correlation between tumour and adjacent normal tissue was observed (Etienne et al, 1995). The goodness of fit of the regression line describing the PMNC–tissue relationship was similar for colorectal tumours ($r^2 = 0.45$) and liver ($r^2 = 0.31$), supporting in part the continued use of PMNCs for monitoring DPD (Chazal et al, 1996). This relationship was also similar to that described between PMNC DPD activity and 5-FU systemic clearance ($r^2 = 0.51$) (Fleming et al, 1992). However, the large degree of variation in tumour activity that was unaccounted for by PMNC activity and the identification of patients with normal PMNC activity but very low liver DPD activity (Stephan et al, 1995) suggest that more accurate alternatives are needed before a high level of confidence can be placed on the widespread use of this approach for targeting 5-FU therapy. The recent identification of mutations in the DPD gene that encode low activity will assist in the prospective identification of patients at high risk for toxicity, but will not account for the large variation in DPD activity in patients with wild-type DPD (Vreken et al, 1996; Wei et al, 1996). Alternative approaches, such as quantitative reverse transcription polymerase chain reaction, are feasible and may offer more insight into the regulation of this protein.

### ACKNOWLEDGEMENTS

These studies could not have been accomplished without the expert assistance of the surgical theatre, oncology department and pathology staffs. We are especially grateful to the Ward 50 phlebotomists for their efforts. This work was supported by a University of Aberdeen Medical Faculty Award and a grant from the Aberdeen Royal Hospitals Trust Endowments.

© Cancer Research Campaign 1998

### REFERENCES

Beck A, Etienne M-C, Cheradame S, Fischel JL, Formento P, Renee N and Milano G (1994) A role for dihydropyrimidine dehydrogenase and thymidylate synthase in tumor sensitivity to fluorouracil. *Eur J Cancer* **30A**: 1517–1522

Bradford MM (1976) A rapid method for the quantitation of microgram quantities of protein utilising the principle of protein-dye binding. *Anal Biochem* **72**: 248–254

Cao SS, Rustum YM and Spector T (1994) 5-Ethynyluracil (776C85) – modulation of 5-fluorouracil efficacy and therapeutic index in rats bearing advanced colorectal carcinoma. *Cancer Res* **54**: 1507–1510

Chazal M, Etienne M-C, Renee N, Bourgeon A, Richelme H and Milano G (1996) Link between dihydropyrimidine dehydrogenase activity in peripheral blood mononuclear cells and liver. *Clin Cancer Res* **2**: 507–510

Etienne MC, Cheradame S, Fischel JL, Formento P, Dassonville O, Renee N, Schneider M, Thyss A, Demard F and Milano G (1995) Response to fluorouracil therapy in cancer patients: the role of tumoral dihydropyrimidine dehydrogenase activity. *J Clin Oncol* **13**: 1663–1670

Fleming RA, Milano G, Thyss A, Etienne M-C, Renee N, Schneider M and Demard F (1992) Correlation between dihydropyrimidine dehydrogenase activity in peripheral mononuclear cells and systemic clearance of fluorouracil in cancer patients. *Cancer Res* **52**: 2899–2902

Hammond AH and Fry JR (1990) The in vivo induction of rat hepatic cytochrome P450-dependent enzyme activities and their maintenance in culture. *Biochem Pharmacol* **40**: 637–642

Harris BE, Song R, Soong S and Diasio RB (1990) Relationship between dihydropyrimidine dehydrogenase activity and plasma 5-fluorouracil levels with evidence for circadian variation of enzyme activity and plasma drug levels in cancer patients receiving 5-fluorouracil by protracted continuous infusion. *Cancer Res* **50**: 197–201

Harris BE, Carpenter JT and Diasio RB (1991) Severe 5-fluorouracil toxicity secondary to dihydropyrimidine dehydrogenase deficiency. *Cancer* **68**: 499–501

Heggie GD, Sommadosi J-P, Cross DS, Huster WJ and Diasio RB (1987) Clinical pharmacokinetics of 5-fluorouracil and its metabolites in plasma, urine, and bile. *Cancer Res* **47**: 2203–2206

Ho DH, Townsend L, Luna MA and Bodey GP (1984) Distribution and inhibition of dihydrouracil dehydrogenase activities in human tissues using 5-fluorouracil as a substrate. *Anticancer Res* **6**: 781–784

Johnston PG, Lenz H-J, Leichman CG, Danenberg KD, Allegra CJ, Danenberg PV and Leichman L (1995) Thymidylate synthase gene and protein expression correlate and are associated with response to 5-fluorouracil in human colorectal and gastric tumors. *Cancer Res* **55**: 1407–1412

Kamm YJL, Rietjens IMC, Vervoort J, Heerschap A, Rosenbusch G, Hofs HP and Wagener DJT (1994) Effect of modulators on 5-fluorouracil metabolite patterns in murine colon carcinoma determined by in vitro $^{19}F$ nuclear magnetic resonance spectroscopy. *Cancer Res* **54**: 4321–4326

Lu Z-H, Zhang R and Diasio RB (1995) Population characteristics of hepatic dihydropyrimidine dehydrogenase activity, a key metabolic enzyme in 5-fluorouracil chemotherapy. *Clin Pharmacol Ther* **58**: 512–522

McKay JA, Murray GI, Weaver RJ, Ewen SWB, Melvin WT and Burke MD (1993) Xenobiotic metabolising enzyme expression in colonic neoplasia. *Gut* **34**: 1234–1239

McMurrough J and McLeod HL (1996) Analysis of the dihydropyrimidine dehydrogenase polymorphism in a British population. *Br J Clin Pharmacol* **41**: 425–427

Murray GI, Taylor MC, McFadyen MCE, McKay JA, Greenlee WF, Burke MD and Melvin WT (1997) Tumor-specific expression of cytochrome P450 CYP1B1. *Cancer Res* **57**: 3026–3031

Naguib FNM, el Kouni MH and Cha S (1985) Enzymes of uracil catabolism in normal and neoplastic human tissue. *Cancer Res* **45**: 5405–5412

Naguib FNM, Hao SN and el Kouni MH (1994) Potentiation of 5-fluorouracil efficacy by the dihydrouracil dehydrogenase inhibitor, 5-benzyloxybenzyluracil. *Cancer Res* **54**: 5166–5170

Smitkamp-Wilms E, Giaccone G, Pinedo HM, van der Laan BFAM and Peters GJ (1995) DT-diaphorase activity in normal and neoplastic human tissues: an indicator for sensitivity to bioreductive agents? *Br J Cancer* **72**: 917–921

Stephan F, Etienne M-C, Wallays C, Milano G and Clergue F (1995) Depressed hepatic dihydropyrimidine dehydrogenase activity and fluorouracil-related toxicities. *Am J Med* **99**: 685–688

Vreken P, van Kuilenburg ABP, Meinsma R, Smit GPA, Bakker HD, de Abreu RA and van Gennip AH (1996) A point mutation in an invariant splice donor site leads to exon skipping in two unrelated Dutch patients with dihydropyrimidine dehydrogenase deficiency. *J Inher Metab Dis* **19**: 645–654

Wang F-S, Aschele C, Sobrero A, Chang Y-M and Bertino JR (1993) Decreased folylpolyglutamate synthetase expression: a novel mechanism of fluorouracil resistance. *Cancer Res* **53**: 3677–3680

Wei X, McLeod HL, McMurrough J, Gonzalez FJ and Fernandez-Salguero P (1996) Molecular basis of the human dihydropyrimidine dehydrogenase deficiency and 5-fluorouracil toxicity. *J Clin Invest* **98**: 610–615

© Cancer Research Campaign 1998